# Three-Dimensional Cutting Force Model and Experimental Study of Diamond Bead Wire Saw Cutting of Loaded Coal

**DOI:** 10.3390/ma19081496

**Published:** 2026-04-08

**Authors:** Shuqing Li, Chenhui Lv, Yunlai Qian, Minghao Yi, Yihong Yang, Zhu Tang, Xiangtao Huang, Tianzhe Zhao

**Affiliations:** 1 School of Resources & Environment and Safety Engineering, Hunan University of Science and Technology, Xiangtan 411201, China; lsq_hnust@163.com (S.L.); 19527407561@163.com (Y.Q.); yangyihong0103@outlook.com (Y.Y.); 15273287889@163.com (X.H.); 17725165968@163.com (T.Z.); 2 School of Mines and Civil Engineering, Liupanshui Normal University, Liupanshui 553004, China; 19967223384@163.com

**Keywords:** diamond bead wire saw, wire saw cutting seam, loaded coal body, cutting force model, cutting experiment

## Abstract

**Highlights:**

**What are the main findings?**

- A theoretical model for three-dimensional cutting force in diamond bead wire cutting of loaded coal bodies is established, enabling prediction under underground stress conditions.
- The stress state of the coal body alters the cutting mechanism, promoting a transition from plastic to brittle deformation and expanding the cutting contact zone.
- Under the condition of coal loading, the expansion of the cutting range dominates, leading to a net increase in cutting force despite the presence of deformation mode changes that partially reduce it.

**Abstract:**

Diamond bead wire saw cutting technology for coal seams is an effective approach for relieving pressure and enhancing permeability by forming internal slits within a coal seam. However, the current lack of solid theoretical guidance for cutting force models in loaded coal bodies makes it difficult to accurately predict cutting forces under underground conditions. This study established a three-dimensional cutting force model for wire saw cutting of loaded coal bodies. At the same time, comparative experiments were conducted using a self-developed experimental apparatus for cutting loaded coal with a wire saw. The research findings indicate that, during wire saw cutting of loaded coal bodies, increasing the cutting depth of the cutting force of a single abrasive grain by changing the load on both sides of the coal body changes the removal patterns of abrasive particles at different orientations on a single bead. This leads to a shift from plastic deformation to brittle deformation while widening the cutting contact area on both sides of the wire saw. The interaction of these aspects changes the cutting force exerted by the wire saw on loaded coal bodies. The experimental results revealed that under high-load conditions, the cutting force on the coal further increased during wire saw cutting. This suggests that the expansion of the cutting range plays a more significant role in increasing the cutting force than does the decrease resulting from changes in removal mode. These findings offer valuable theoretical insights for the process design of wire saw coal cutting technology.

## 1. Introduction

Diamond bead wire saws (wire saw) have been widely applied in stone cutting and processing, mining and quarrying, metal cutting, and construction engineering. In recent years, wire saw cutting technology for coal seams has attracted significant attention as an effective method for stress relief and permeability enhancement [1,2,3]. It is well known that wire saw cutting technology has traditionally targeted high-hardness rocks or structural components, primarily in surface-level open-pit operations, without considering the influence of ground stress. In contrast, coal seam wire saw cutting technology targets soft coal layers, where the cutting process is affected by ground stress, specifically the loading pressure exerted from both sides during cutting. Given the underground mining context, developing a cutting force model specifically for the wire saw when operating on loaded coal seams is crucial. This model directly affects critical aspects of coal seam cutting operations, including the selection of wire saw machines, saw blades, and supporting equipment; protection against wire breakage through wire tension calculations; and power supply requirements. Therefore, research on the cutting force of wire saw-cutting loaded coal bodies is highly important for improving the design of diamond wire saw-cutting coal seams and improving the cutting efficiency.

During the cutting of coal seams with a wire saw, the drive motor rotates the drive wheel at high speed, which in turn drives the tensioned wire saw to move cyclically, primarily in the tangential direction. Simultaneously, continuous tension is maintained on the wire through the coordinated motion of the cutting machine and the guiding wheels, thereby realizing the feed motion. During the tensioning process, the wire saw closely adheres to the coal body and contacts it under a certain pressure. Subsequently, through the motion of the saw, frictional and cutting forces are generated, forming a cutting slit within the coal mass. This composite forming motion enables the cutting of the coal body. The wire saw is a flexible cutting tool, and its cutting action is essentially abrasive machining. The abrasive action of the wire saw results from the cumulative effect of numerous micro-cuts produced by the high-speed movement of diamond abrasive grains, which act as micro-cutting tools. At present, numerous scholars have extensively researched the cutting mechanisms of the wire saw. Key viewpoints include the fact that the wire saw operates at a high cutting line speed, whereas the pressure exerted by individual diamond grains on the rock is relatively small; under repeated high-speed grinding and scraping by the wire saw, the dominant rock fragmentation mode is Hertzian fracture; and factors such as wire saw linear speed, feed rate, number of abrasive grains per unit area on the bead surface, effective cutting coefficient of abrasive grains, and the angle at the base of rock chips significantly influence rock cutting behavior [4,5,6].

Regardless of loading conditions, the modeling of the cutting forces for a single abrasive grain has been a central area of investigation. Li et al. [7] examined the mechanism of cutting force generation by individual diamond grains on electroplated diamond wire saws. Based on brittle material fracture theory, they analyzed the formation of cutting and frictional forces between wafers and abrasive grains during SiC single-crystal wafer cutting and formulated a force model for single abrasive grains. Wang et al. [8] developed a theoretical model for forces at the nano- and micro-scale during abrasive engagement with single-crystal SiC wire saws that is applicable to arbitrary scratching angles. By integrating the abrasive geometry, interfacial friction, scale effects, and elastic recovery, this model closely matches the experimental data and reveals the mechanisms by which process variables influence scratching forces. Meng et al. [9] reported that the material removal mode of a single abrasive grain depends on its cutting depth: below a critical depth, removal shifts from brittle to plastic. Wang et al. [10] introduced a single-abrasive-grain cutting force calculation approach that incorporates frictional forces by combining brittle and plastic removal theories. They analyzed how process parameters and saw parameters influence the cutting force and ultimately derived a mathematical expression describing the cutting force based on these parameters using a new computational approach. Wu and Melkote [11] employed an extended finite element method to investigate the transition from plastic to brittle cutting mechanisms during monocrystalline silicon processing. They found that, when scratching monocrystalline silicon surfaces with diamond styluses, the critical cutting depth for plastic-to-brittle transition depends on the stylus tip geometry, friction coefficient, and static pressure.

Based on the aforementioned research, some scholars have also examined how ultrasonic vibration affects cutting forces during cutting, establishing cutting force models for ultrasonic wire cutting. Wang et al. [12] systematically clarified the mechanism by which ultrasonic-assisted diamond wire sawing (UAWS) reduces the cutting force. This was achieved by developing a theoretical model of lateral vibration and single-abrasive-particle impact load for UAWS, combined with finite element simulation and multi-abrasive-particle distribution analysis. Experimental validation showed an average error of 7.50% between theoretical and measured cutting forces, with UAWS reducing workpiece surface roughness by 4.3–29.7% compared with conventional wire sawing (CWS). Zhang et al. [13] formulated a mathematical representation of cutting forces in electroplated diamond wire saw ultrasonic cutting grounded in impulse theory and vibration machining theory using the superposition principle. Comparative experimental studies were carried out between ultrasonic-assisted cutting and traditional cutting forces. Li et al. [14] investigated diamond wire sawing of hard and brittle materials with ultrasonic vibration assistance. By analyzing the forced vibration of the wire saw, they determined the conditions required to achieve flexible rotary point cutting under transverse ultrasonic excitation and characterized the machining state via characteristic functions. The actual cutting process often requires the participation of most beads and abrasive grains within the cutting arc zone. Liedke and Kuna [15] proposed a theoretical framework to describe the overall mechanical behavior during wire sawing, detailing the effects of the cutting process parameters and geometric relationships on the winding pressure and the profile of the resulting wire bow. In addition, many researchers have examined the effects of various cutting factors on cutting forces on the basis of cutting force models. Cheng et al. [16] formulated and coupled a sawing force model with a finite element model, revealing stress distribution patterns dominated by thermomechanical coupling during multi-wire sawing of polycrystalline silicon and clarifying the specific influence mechanisms of process parameters and wafer dimensions on the magnitude and distribution of coupled stresses, which are predominantly thermal stresses. This study offers a theoretical foundation for comprehending sawing damage from a mechanical perspective and for optimizing processes to increase wafer strength. Huang et al. [17] carried out cutting tests on A-face and C-face sapphire using an oscillating diamond wire saw and systematically investigated the impact of machining conditions and sapphire crystalline orientation on cutting forces. The findings indicate that during the slicing of sapphire, the crystallographic orientation, wire velocity, and feed velocity significantly affect the tangential cutting force, which shows a strong linear correlation with material removal rates (MRRs). Tang et al. [18] generated simplified irregular polyhedral abrasive particles using a random spatial planar sphere method combined with a stochastic procedure to determine the positions of the abrasive particles in 3D space and constructed a three-dimensional representation of the diamond wire saw surface that more closely approximates actual conditions. They examined how the feed rate and wire saw speed influence the cutting force and extended this multi-abrasive-particle cutting force model to the macroscale regime of wire cutting based on contact arc length. The accuracy of the model was validated through 4H-SiC machining trials.

The cutting force models established in the aforementioned studies consider only the pressure actively applied to the material by the wire saw during conventional cutting and do not address the influence of lateral loading pressures acting on the wire saw. Existing cutting force models cannot explain the variation patterns of cutting forces or the parameter influence relationships during wire saw cutting of loaded coal bodies. Therefore, this study incorporates the loading conditions on both sides of the coal body. By defining the active-passive force relationship during wire saw cutting and clarifying the plastic-brittle transition mechanism of single abrasive grain removal from coal, a force model for individual beads under coal loading is established. On this basis, a cutting force equation for the entire cutting arc zone is derived. The impacts of the wire saw cutting parameters and coal body parameters on the cutting force are then analyzed. Finally, the model’s predictive ability is confirmed through loaded coal machining tests using a self-developed experimental apparatus for wire saw cutting of loaded coal bodies. The proposed cutting force model effectively predicts the magnitude of the cutting force during wire saw cutting of loaded coal, providing a theoretical foundation for the design of underground coal seam cutting processes using wire saw.

## 2. Three-Dimensional Cutting Force Model

### 2.1. Single-Grain Cutting Force Model

The cutting force exerted by individual abrasive grains is critical for both material removal and the prediction of cutting forces during wire saw cutting. When a wire saw cuts loaded coal bodies, the total cutting force is the sum of the forces acting on all diamond abrasive grains participating in the cutting process. Therefore, analysis of the force model for individual grains is essential. In current studies of cutting forces for bonded abrasive wire saws, it is generally assumed that the abrasive grains on the bead surface are sharp conical particles. Moreover, the cutting area of the processed coal body is much smaller than the cutting life of the bead wire. Accordingly, the effects of abrasive grain wear are not considered in this study.

**Figure 1:**
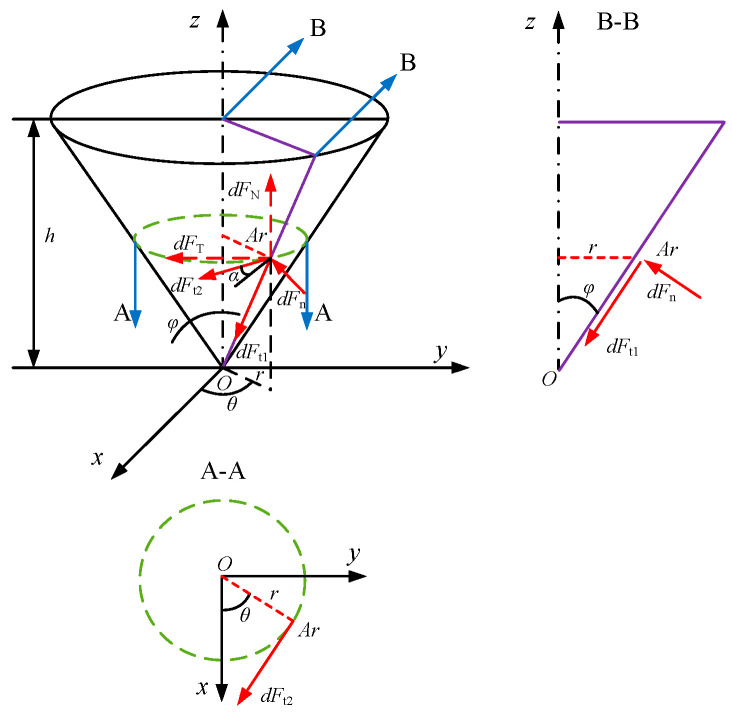
Schematic diagram of forces acting on a single abrasive grain.

The interaction between abrasive particles and coal during wire saw cutting is similar to that in grinding processes, with the cutting force mainly composed of cutting deformation force and friction deformation force [19]. This study adopts a cutting force calculation method proposed by Zhang et al. [20] that incorporates the friction force component. The force distribution between the abrasive and coal during cutting is shown in Figure 1. *A*_r_ denotes an infinitesimal element region on the conical contact surface between the abrasive and coal, with an area of *d*s; *dF*_n_ is the normal force component perpendicular to *A*_r_; *dF*_t2_ is the tangential force component tangent to the circular cross-section of the conical abrasive within the *A*_r_ region; and *dF*_t1_ is the tangential force component within the *A*_r_ region that is perpendicular to *d*F_t2_. The angle α is the angle between the tangential force component *dF*_t2_ and the resultant of the tangential force components *dF*_t1_ and *dF*_t2_. *φ* is the half-cone angle of the abrasive, and *h* is the cutting depth.

In the model shown in Figure 1, the normal component d*F*_n_ is proportional to the area ds of the infinitesimal element region *A*_r_ and can be expressed as:

(1) $$dF_{n}=\xi ds$$

In this equation, *ξ* is a constant related to the properties of the coal body and the material removal mode.

Based on the geometric relationship illustrated in Figure 1, we obtain:

(2) $$\left\{\begin{array}{l} dF_{t1}=\mu_{a}\xi \sin\alpha ds\\ dF_{t2}=\mu_{a}\xi \cos\alpha ds\\ \sin\alpha =\frac{\sin\varphi \sin\theta }{\sqrt{1-\sin^{2}\theta \cos^{2}\varphi }}\\ \cos\alpha =\frac{\cos\theta }{\sqrt{1-\sin^{2}\theta \cos^{2}\varphi }} \end{array}\right.$$

Among these, *μ*_α_ represents the interfacial friction coefficient, which is typically taken as 0.2 under water-lubricated conditions [21,22].

The forces acting within region *A*r can be decomposed into the normal cutting force *dF*_Ni_ and the tangential cutting force *dF*_Ti_ along the abrasive grain.

(3) $$\left\{\begin{array}{l} dF_{Ni}=dF_{n}\sin\varphi -dF_{t1}\cos\varphi \\ dF_{Ti}=dF_{n}\cos\varphi \sin\theta +dF_{t1}\sin\varphi \sin\theta +dF_{t2}\cos\theta \end{array}\right.$$

The area of *ds* in polar coordinates can be expressed as:

(4) $$ds=\sqrt{1+\cot^{2}\varphi }\cdot rd\theta dr$$

The normal cutting force *dF*_Ni_ and tangential cutting force *dF*_Ti_ acting on the abrasive grain can be calculated as:

(5) $$\left\{\begin{array}{l} F_{Ni}=2\int_{0}^{R}\int_{0}^{\frac{\pi }{2}}[\xi \sin\varphi -\mu_{a}\xi \frac{\sin\varphi \cos\varphi \sin\theta }{\sqrt{1-\sin^{2}\theta \cos^{2}\varphi }}]\sqrt{1+\cot^{2}\varphi }\cdot rd\theta dr\\ F_{Ti}=2\int_{0}^{R}\int_{0}^{\frac{\pi }{2}}[\xi \cos\varphi \sin\theta -\mu_{a}\xi \sqrt{1-\sin^{2}\theta \cos^{2}\varphi }]\sqrt{1+\cot^{2}\varphi }\cdot rd\theta dr \end{array}\right.$$

The solution of Equation (5) can be further expressed as:

(6) $$\left\{\begin{array}{l} F_{Ni}=\xi h^{2}\tan\varphi \cdot [\frac{\pi }{2}-\mu_{a}\cos\varphi K_{N}]\\ F_{Ti}=\xi h^{2}\tan\varphi \cdot [\cos\varphi +\frac{\mu_{a}K_{T}}{\sin\varphi }] \end{array}\right.$$

In this equation, *K*_N_ and *K*_T_ are defined as:

(7) $$\left\{\begin{array}{l} K_{N}=\int_{0}^{\frac{\pi }{2}}\frac{\sin\theta }{\sqrt{1-\sin^{2}\theta \cos^{2}\varphi }}d\theta \\ K_{T}=\int_{0}^{\frac{\pi }{2}}\sqrt{1-\sin^{2}\theta \cos^{2}\varphi }d\theta \end{array}\right.$$

The half-cone angle of abrasive grains typically ranges from 45° to 60°. When *φ* is taken as 55°, *K*_N_ = 1.1382 and *K*_T_ = 1.4323.

During the cutting of hard and brittle materials, plastic and brittle removal modes coexist, with plastic cutting generally associated with higher cutting forces. It is known that plastic removal can occur at lower cutting depths. Bifano et al. [23] conducted quasi-static scratch experiments using microindentation and found that the critical depth *d*_c_ for the transition from plastic to brittle removal can be expressed as:

(8) $$d_{c}=0.15\cdot \frac{E}{H}{\left(\frac{K_{c}}{H}\right)}^{2}$$

In this equation, *K*_C_ denotes the fracture toughness of the material, expressed in MPa · m^1/2^. The fracture toughness *K_C_* of the coal specimen was measured using three-point bending tests; E is the elastic modulus of the coal body, expressed in GPa; and H is the microhardness of the coal body (Vickers hardness), expressed in GPa.

In the brittle removal mode, the normal cutting force of a single abrasive grain can be expressed as [24]:

(9) $$F_{Nib}=\alpha_{1}h^{2}\tan^{2}\varphi \cdot H$$

In this equation, *α*_1_ is a dimensionless constant, with *α*_1_ = π/2.

According to Equation (6), the ratio of the tangential cutting force to the normal cutting force for a single abrasive grain can be expressed as:

(10) $$\frac{F_{Ti}}{F_{Ni}}=\frac{\cot\varphi +\mu_{a}K_{T}}{\frac{\pi }{2}\sin\varphi -\mu_{a}\sin\varphi \cos\varphi K_{N}}$$

Therefore, in the brittle removal mode, the tangential cutting force of a single abrasive grain can be further expressed as:

(11) $$F_{Tib}=\frac{\cot\varphi +\mu_{a}K_{T}}{\frac{\pi }{2}\sin\varphi -\mu_{a}\sin\varphi \cos\varphi K_{N}}\alpha_{1}h^{2}\tan^{2}\varphi \cdot H$$

In Equation (1), the constant *ξ* [25] can be regarded as the yield stress *Y* of the material under the plastic removal mode. The yield stress *Y* can be calculated using Equation (12) [26].

(12) $$Y=\frac{E}{3(1-v)(\beta^{3})-2(1-2\upsilon )}$$

In this equation, *ν* represents the Poisson’s ratio of the workpiece, and *β* denotes the relative plastic dimension of the material indented by the abrasive grain, specifically the ratio of the radius of the hemispherical plastic zone to the radius of the hemispherical indentation. For materials such as coal with relatively low strength, the plastic zone is comparatively large.

Therefore, under the plastic removal mode, the normal cutting force and tangential cutting force exerted by a single abrasive grain can be expressed as:

(13) $$\left\{\begin{array}{l} F_{Nid}=Yh^{2}\tan\varphi \cdot [\frac{\pi }{2}-\mu_{a}\cos\varphi K_{N}]\\ F_{Tid}=Yh^{2}\tan\varphi \cdot [\cot\varphi +\frac{\mu_{a}K_{T}}{\sin\varphi }] \end{array}\right.$$

### 2.2. Single Bead Saw Cutting Force Model

**Figure 2:**
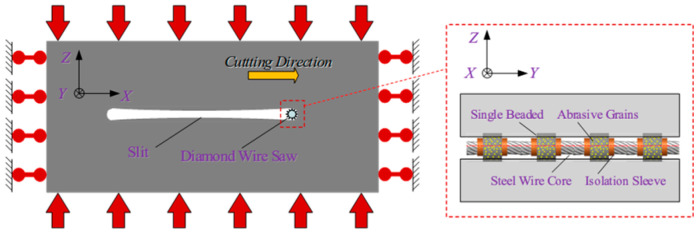
Schematic of the directional cutting of coal seams by a wire saw and the contact conditions between the saw and the coal body.

After coal seams subjected to wire saw cutting are initially slitted, the seams undergo displacement toward the slit interior under in situ stress. This displacement of both the roof and floor coal masses at the cutting face often increases the contact area with the wire saw. Figure 2 illustrates the strike direction of the coal seam being cut and the contact conditions between the wire saw and the coal body.

**Figure 3:**
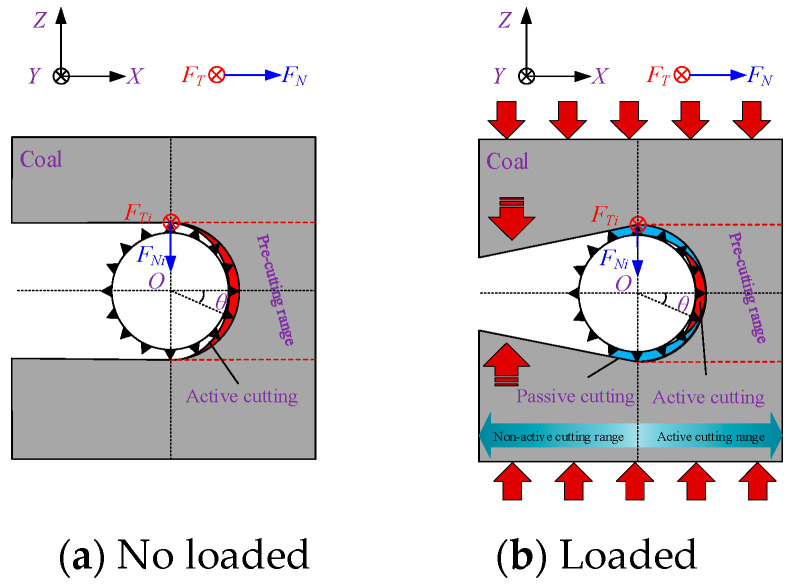
Force conditions before and after loading for abrasive grains at different orientations.

Therefore, when calculating the cutting force of the entire bead string, it is necessary to account for the increased contact area caused by coal body loading and to consider how coal removal patterns in different contact regions change under increased loading conditions. Studies show that under non-loaded conditions, the cutting beads of the wire are concentrated on the half-surface in the cutting direction. The cutting depth of abrasive grains varies with azimuth angle, as shown in Figure 3a. The cutting depth of grains near the cutting direction (right side) is greater than that of grains on the upper and lower sides of the wire saw. In this case, coal removal is entirely driven by the force actively applied by the wire saw, which presses abrasive particles into the coal. The wire saw actively cuts the coal as it advances in the cutting direction. When the wire saw cuts loaded coal, displacement of the upper and lower coal layers alters the contact configuration with the wire saw, increasing the contact area with the coal, as shown in Figure 3b. At this stage, the coal in the pre-cut zone ahead of the cutting face acts as a coal pillar. The upward- or downward-displaced coal contacts the cutting wire saw and presses the coal to be cut into the abrasive particles, resulting in passive cutting by the abrasive grains.

The above analysis indicates that calculating the normal and tangential cutting forces over the entire contact arc region requires analysis of cutting forces under different abrasive particle removal modes. According to the study by Li et al. [27], the volume of material removed by a single abrasive particle differs under different removal modes. During plastic removal, the removal rate *V*_0_ of a single particle can be expressed by Equation (14).

(14) $$V=\frac{1}{2}bhv_{s}=h^{2}v_{s}\tan\varphi$$

In this equation, *b* represents the width of the abrasive grain in the cutting material.

**Figure 4:**
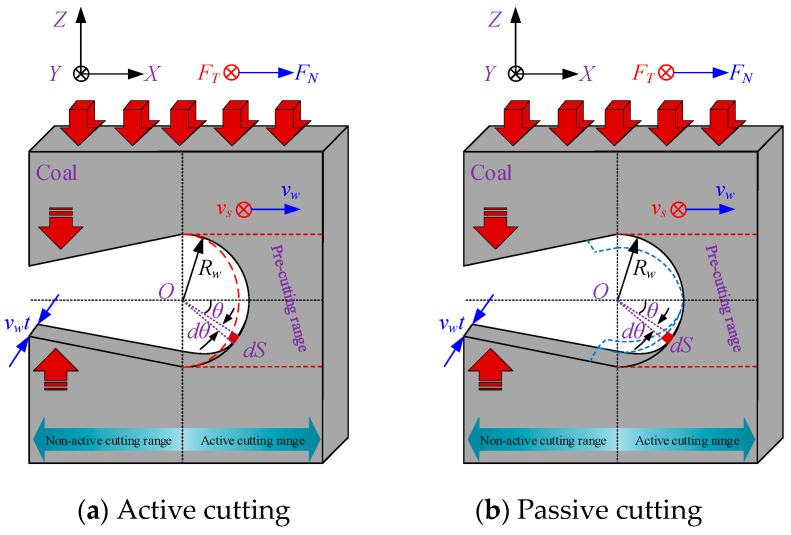
Schematic of the wire saw in contact with the coal body.

Figure 4a shows the active cutting range of the wire saw in contact with the coal body. *R*_w_ denotes the envelope radius of the wire saw at the bead connection point; the arc length *dS* = *R*_w_*dθ* corresponds to the angle *dθ*, and the coal contact area *dA* = *L*_w_*dS* corresponds to the arc length *dS*, where *L*_w_ is the contact length between the wire saw and the coal body, calculated as the length of a single bead. If *C* is the density of abrasive grains, the number of grains *N*_C_ within the area *dA* is *N*_C_ = *C·dA*.

Therefore, the volume of coal removed per unit time by the wire saw is *V*_W_:

(15) $$V_{W}=V_{0}Cv_{s}dS=C{h_{1}}^{2}{v_{s}}^{2}\tan\varphi dS$$

In this equation, *h*_1_ represents the abrasive cutting depth during active plastic cutting.

At the same time, the cutting surface of the wire saw advances at a feed rate of *v*_w_. The volume of coal removed per unit time can also be expressed as:

(16) $$V_{W}=v_{s}v_{w}\cos\theta dS$$

Combining Equations (15) and (16) yields the cutting depth *h*_1_ of the abrasive grain at the arc length *dS* during active cutting.

(17) $$h_{1}=\sqrt{\frac{v_{w}\cos\theta }{Cv_{s}\tan\varphi }},\left(-\frac{\pi }{2}<\theta <\frac{\pi }{2}\right)$$

Similarly, it yields the cutting depth *h*_2_ of the abrasive grain at the arc length *dS* during passive cutting of the coal body.

(18) $$h_{1}=\sqrt{\frac{v_{m}\cos\left(\theta -\frac{\pi }{2}\right)}{Cv_{s}\tan\varphi }}=\sqrt{\frac{v_{m}\sin\theta }{Cv_{s}\tan\varphi }},\left(-\frac{\pi }{2}<\theta <\frac{\pi }{2}\right)$$

In this equation, *v*_m_ represents the relative velocity between the vertical wire saw and the coal body above and below it, which can be monitored experimentally.

**Figure 5:**
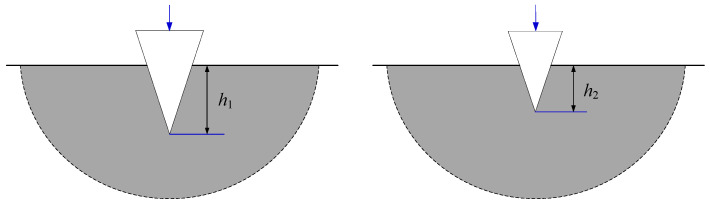
Abrasive cutting depth under different cutting methods.

Additionally, the abrasive grains used in the cutting process are conical in shape, as shown in Figure 5.

Therefore, under the combined effects of active and passive cutting, the cutting depth *h* of the abrasive grain at the arc length dS in the plastic removal zone is given by:

(19) $$h=\eta_{d}\left(h_{1}+h_{2}\right)$$

In this equation, *η*_d_ is the particle depth coefficient during plastic removal and is related to *θ*.

Substituting Equation (19) into Equation (13) yields the expressions for the normal cutting force and tangential cutting force of a single abrasive particle:

(20) $$\left\{\begin{array}{l} F_{Nid}=\frac{Y{\eta^{2}}_{d}}{Cv_{s}}{\left(\sqrt{v_{w}\cos\theta }+\sqrt{v_{m}\sin\theta }\right)}^{2}\tan\varphi \cdot \left(\frac{\pi }{2}-\mu_{a}\cos\varphi K_{N}\right)\\ F_{Tid}=\frac{Y{\eta^{2}}_{d}}{Cv_{s}}{\left(\sqrt{v_{w}\cos\theta }+\sqrt{v_{m}\sin\theta }\right)}^{2}\tan\varphi \cdot \left(\cot\varphi +\frac{\mu_{a}K_{T}}{\sin\varphi }\right) \end{array}\right.,\left(-\frac{\pi }{2}<\theta <\frac{\pi }{2}\right)$$

**Figure 6:**
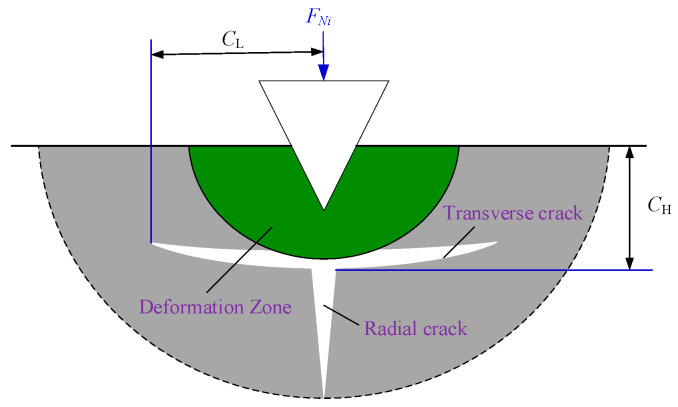
Cracks in brittle materials.

According to the critical depth criterion for removal mode transition given by Equation (8), when the cutting depth of abrasive particles increases, the removal mode of the coal body changes to brittle removal, resulting in the cracks shown in Figure 6.

The transverse crack depth *C*_H_ and transverse crack length *C*_L_ were obtained by Marshall et al. [28] and Xiao et al. [29], as given in Equation (21):

(21) $$\left\{\begin{array}{l} C_{L}=C_{1}{\left(\frac{1}{\tan\varphi }\right)}^{5/12}{\left(\frac{E^{3/4}}{HK_{C}{\left(1-\upsilon^{2}\right)}^{2}}\right)}^{1/2}{\left(F_{Ni}\right)}^{5/8}\\ C_{H}=C_{1}{\left(\frac{1}{\tan\varphi }\right)}^{1/3}\frac{E^{1/2}}{H}{\left(F_{Ni}\right)}^{1/2} \end{array}\right.$$

In this equation, *K*_c_ represents the fracture toughness, expressed in MPa·m^1^ᐟ^2^; *E* denotes the elastic modulus of the coal body, expressed in GPa; and *C*_1_ is a dimensionless constant independent of both material and indenter system, with *C*_1_ = 0.226.

At this stage, the coal removal rate per abrasive grain during wire saw cutting is:

(22) $$V_{0}=2C_{L}V_{H}v_{s}$$

The volume of coal removed per unit time within the differential arc *dS* is:

(23) $$V_{W}=V_{0}Cv_{s}dS=2C_{L}V_{H}v_{s}^{2}dS$$

Combining Equations (16)–(18) and (23), the penetration depth *h*_1_ of abrasive particles into the coal body during active cutting in the brittle removal mode can be obtained as:

(24) $$h_{1}={\left(\frac{v_{m}\cos\theta }{2\alpha_{1}^{9/8}Cv_{s}}\cdot \frac{H^{3/8}K_{C}^{1/2}(1-\upsilon^{2})}{C_{1}^{2}\tan\varphi^{3/2}E^{7/8}}\right)}^{4/9},\left(-\frac{\pi }{2}<\theta <\frac{\pi }{2}\right)$$

Similarly, for determining the passive cutting range in the brittle removal mode, the penetration depth *h*_2_ of abrasive particles into the coal body is:

(25) $$h_{2}={\left(\frac{v_{m}\sin\theta }{2\alpha_{1}^{9/8}Cv_{s}}\cdot \frac{H^{3/8}K_{C}^{1/2}(1-\upsilon^{2})}{C_{1}^{2}\tan\varphi^{3/2}E^{7/8}}\right)}^{4/9},\left(-\frac{\pi }{2}<\theta <\frac{\pi }{2}\right)$$

Further, within the active cutting zone, under the combined effects of active and passive cutting, the cutting depth *h* of the abrasive grain at the arc length *dS* in the brittle removal region is given by:

(26) $$h=\eta_{b}(h_{1}+h_{2})$$

In this equation, *η*_b_ is the abrasive depth coefficient during brittle removal and is related to *θ*.

Substituting Equation (26) into Equations (9) and (11) yields the normal cutting force and tangential cutting force of a single abrasive grain as follows:

(27) $$\left\{\begin{array}{l} F_{Nib}=\eta_{b}^{2}{\left({\left(v_{w}\cos\theta \right)}^{4/9}+{\left(v_{m}\sin\theta \right)}^{4/9}\right)}^{2}\cdot {\left(\frac{K_{C}^{1/2}(1-v^{2})}{2Cv_{s}C_{1}^{2}E^{7/8}}\right)}^{8/9}\cdot {\left(\tan\varphi \right)}^{2/3}H^{4/3}\\ F_{Tib}=\frac{\cot\varphi +\mu_{a}K_{T}}{\frac{\pi }{2}\sin\varphi -\mu_{a}\sin\varphi \cos\varphi K_{N}}\eta_{b}^{2}{\left({\left(v_{w}\cos\theta \right)}^{4/9}+{\left(v_{m}\sin\theta \right)}^{4/9}\right)}^{2}\cdot {\left(\frac{K_{C}^{1/2}(1-v^{2})}{2Cv_{s}C_{1}^{2}E^{7/8}}\right)}^{8/9}\cdot {\left(\tan\varphi \right)}^{2/3}H^{4/3} \end{array}\right.,\left(-\frac{\pi }{2}<\theta <\frac{\pi }{2}\right)$$

Li et al. [30] found that during the active cutting of unloaded workpieces by a wire saw, the normal cutting force in the cutting direction of the wire saw is generally greater within the contact semicircular arc. This causes the cutting depth of abrasive grains into the coal body to typically exceed the critical depth *d*_c_. As a result, abrasive grains near the cutting direction of the wire saw remove coal mainly through brittle removal, whereas grains near both sides of the wire saw exhibit plastic removal. The ratio of abrasive grains causing brittle removal to those causing plastic removal ranges from 1/5 to 1/4, with 1/4 (π/8) adopted in this study. The underlying principle is that when the direction of the normal cutting force of abrasive grains on the bead coincides with the relative motion direction between the wire saw and the workpiece along a straight line, this point marks the initiation of brittle cutting. The brittle removal region then extends outward toward both sides of the contact arc zone by π/8.

**Figure 7:**
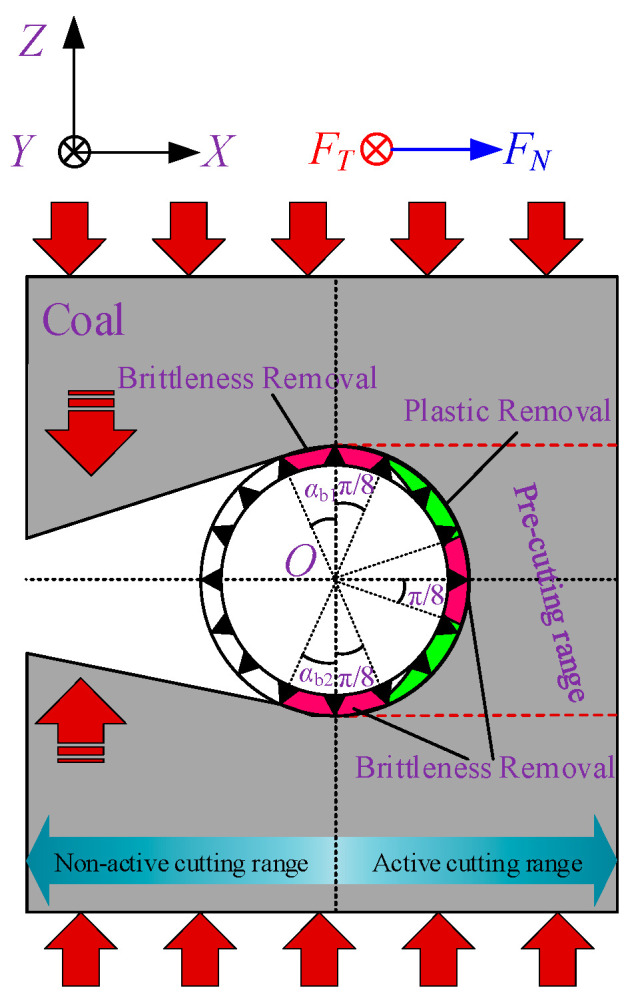
Removal mechanism of abrasive particles in the contact arc zone.

**Figure 8:**
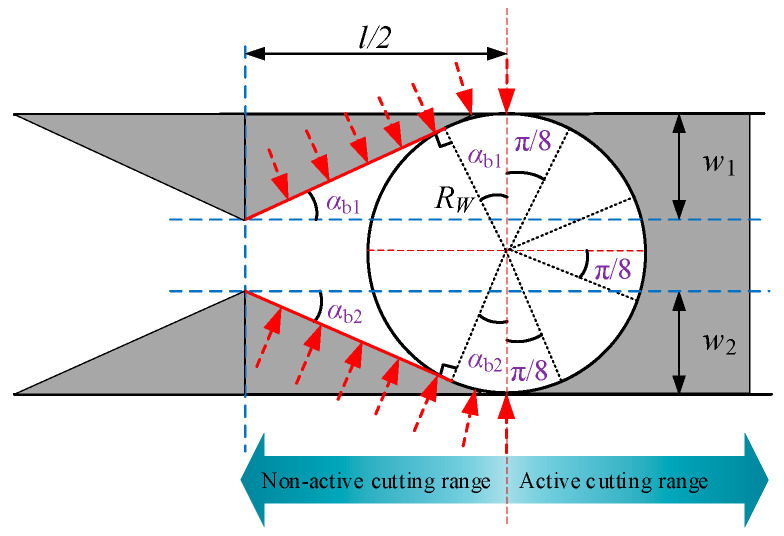
Geometric relationship of the curved removal range for the wire saw.

When the wire saw cuts loaded coal bodies, particularly under actual mining conditions, the coal seam is often subjected to considerable pressure. This causes the normal cutting force acting on the wire saw in the passive cutting range to be much greater than that in the active cutting direction. When considering coal removal behavior in passive cutting, the boundaries between brittle and plastic removal must therefore be redefined. Within the non-active cutting zone, the forces exerted on the wire saw by the coal masses above and below the formed seam both pass through the center of the wire saw cross-section, and the cutting angles are tangential to the wire saw, as shown in Figure 7 and Figure 8. As a result, the contact area between the coal masses above and below the wire saw within the slitted zone is entirely dominated by brittle cutting. This indicates that, within the non-active cutting range, the movement direction of the coal mass coincides with the direction of the abrasive normal force. When calculating the cutting force exerted by abrasive grains on the coal mass, the cutting depth produced by brittle removal in this region is equivalent to the cutting depth at *θ* = 0. At the same time, the wire saw is subjected to vertical pressure from both the upper and lower coal bodies during cutting.

Based on the criteria for distinguishing brittle and plastic removal, within the active cutting range, the ratio of abrasive grains undergoing brittle removal to those undergoing plastic deformation in the coal strata above and below the wire saw along the cutting direction is taken as 1/4. In addition, the coal strata near the direction of the vertical force applied to the wire saw experience brittle removal. Owing to the effect of passive cutting, the removal mode of coal above and below the active cutting range changes from plastic removal to brittle removal.

Based on the above analysis, the cutting force acting on the coal body during wire saw cutting is related not only to the contact area between the wire saw and the coal body but also to the removal modes of the coal body induced by abrasive particles at different orientations. The values of *α*_b1_ and *α*_b2_ can be determined from the geometric relationships shown in Figure 8, where *w*_1_ and *w*_2_ represent the maximum displacement of the coal mass toward the slit space on either side of the slit (displacement at the slit midpoint or closure point), expressed in m, and *l*/2 denotes the distance from the slit midpoint or closure point to the cutting face, expressed in m.

The deflection of the coal mass toward the slit space is calculated using a rigid beam model [3]:

(28) $$w=\frac{ql^{4}}{32Ebh^{3}}$$

In this equation, *q* represents the uniformly distributed load applied on both sides, MPa; *b* denotes the width of the coal beam, m; and *h* indicates the height of the coal beam, m.

An approximate expression can be obtained:

(29) $$\left\{\begin{array}{l} \alpha_{b1}=\arctan\frac{ql^{3}}{16Ebh_{1}^{3}}\\ \alpha_{b2}=\arctan\frac{ql^{3}}{16Ebh_{2}^{3}} \end{array}\right.$$

Based on the above analysis, during the active cutting stage of the wire saw, brittle removal mainly occurs along the cutting direction and within the contact areas on both the upper and lower sides of the wire saw. Plastic removal is mainly concentrated at the oblique upper and lower angular regions relative to the cutting direction of the wire saw.

Therefore, according to Equation (27), the cutting force expression for brittle removal in the non-active cutting range (*θ* > π/2 or *θ* < π/2) can be supplemented.

(30) $$\left\{\begin{array}{l} F_{Nib}={\left(\frac{K_{C}^{1/2}(1-v^{2})}{2C_{1}^{2}E^{7/8}}\right)}^{8/9}\cdot {\left(\tan\varphi \right)}^{2/3}H^{4/3}{\left(\frac{v_{w}}{Cv_{s}}\right)}^{8/9}\\ F_{Tib}=\frac{\cot\varphi +\mu_{a}K_{T}}{\frac{\pi }{2}\sin\varphi -\mu_{a}\sin\varphi \cos\varphi K_{N}}{\left(\frac{K_{C}^{1/2}(1-v^{2})}{2C_{1}^{2}E^{7/8}}\right)}^{8/9}\cdot {\left(\tan\varphi \right)}^{2/3}H^{4/3}{\left(\frac{v_{w}}{Cv_{s}}\right)}^{8/9} \end{array}\right.,\left(\theta <-\frac{\pi }{2}\cup \theta >\frac{\pi }{2}\right)$$

**Figure 9:**
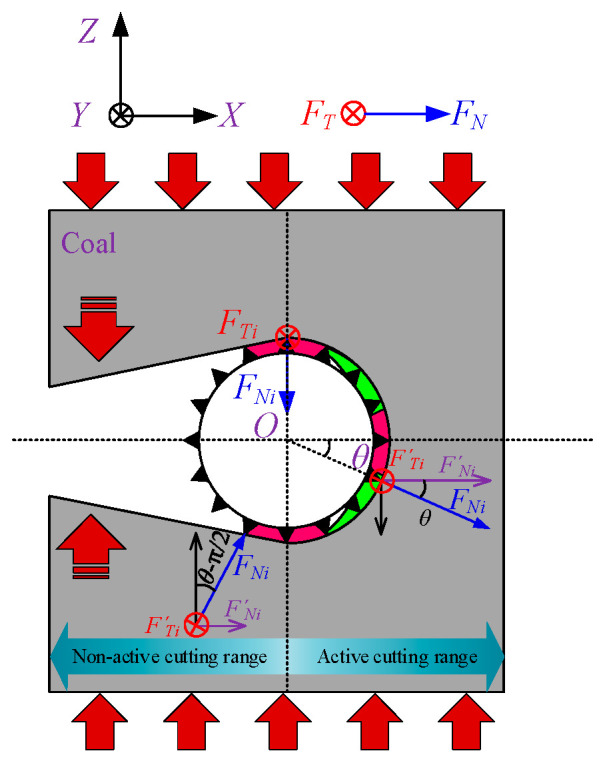
Schematic diagram of normal force decomposition.

Equations (20), (27) and (30) represent the normal and tangential cutting forces of a single abrasive grain. The effective forces exerted by single abrasive grains, *F*_Nib_ and *F*_Nid_, on the coal body are the components along the *X*-axis, which is consistent with the cutting direction of the wire saw. It should be noted that, within the non-active cutting range, part of the internal force associated with stress release is converted into an external force. This force can be transmitted through the wire saw in the cutting direction (positive *X*-direction). The resultant of the component forces exerted by the wire saw on the coal body constitutes the cutting force acting on the loaded coal body, as shown in Figure 9.

Accordingly, the normal cutting force and tangential cutting force exerted by a single abrasive grain on the coal body are given by Equations (31) and (32), respectively.

Plastic Removal:

(31) $$\left\{\begin{array}{l} F_{Nid}^{\prime }=F_{Nib}\cos\theta \\ F_{Tid}^{\prime }=F_{Tib} \end{array}\right.,\left(-\frac{\pi }{2}<\theta <\frac{\pi }{2}\right)$$

Brittleness Removal:

(32) $$\left\{\begin{array}{l} F_{Nid}^{\prime }=F_{Nib}\cos\theta ,\left(-\frac{\pi }{2}<\theta <\frac{\pi }{2}\right)\\ F_{Nid}^{\prime }=F_{Nib}\sin\left(\theta -\frac{\pi }{2}\right),\left(\theta <-\frac{\pi }{2}\cup \theta >\frac{\pi }{2}\right)\\ F_{Tid}^{\prime }=F_{Tib} \end{array}\right.$$

From the above analysis, the cutting force *dF* exerted by abrasive grains on the coal body within the differential area *dA* is equal to the sum of the cutting forces exerted by all abrasive grains *N*_C_ in that area. Therefore, during plastic removal, the normal and tangential cutting forces exerted by abrasive grains on the coal body within the differential area *dA* are, respectively:

(33) $$\left\{\begin{array}{l} dF_{DN}=F_{Nid}^{\prime }N_{C}=F_{Nid}^{\prime }L_{W}R_{W}d\theta \\ dF_{DT}=F_{Tid}^{\prime }N_{C}=F_{Tid}^{\prime }L_{W}R_{W}d\theta \end{array}\right.$$

During brittle removal, the normal and tangential cutting forces within the differential area *dA* are, respectively:

(34) $$\left\{\begin{array}{l} dF_{BN}=F_{Nib}^{\prime }N_{C}=F_{Nib}^{\prime }L_{W}R_{W}d\theta \\ dF_{BT}=F_{Tib}^{\prime }N_{C}=F_{Tib}^{\prime }L_{W}R_{W}d\theta \end{array}\right.$$

Based on the above, by taking the length of *R*_W_ as the length of a single bead, the expressions for the normal cutting force *F*_N_ and tangential cutting force *F*_T_ acting on a single bead are:

(35) $$F_{N}=\left\{\begin{array}{l} \int_{-\frac{\pi }{2}-\alpha_{b2}}^{-\frac{\pi }{2}}\sin\left(\theta -\frac{\pi }{2}\right){\left(\frac{K_{C}^{1/2}(1-v^{2})}{2C_{1}^{2}E^{7/8}}\right)}^{8/9}\cdot {\left(\tan\varphi \right)}^{2/3}H^{4/3}{\left(\frac{v_{w}}{Cv_{s}}\right)}^{8/9}L_{W}R_{W}d\theta \\ \int_{-\frac{\pi }{2}}^{-\frac{3\pi }{8}}\cos\theta \cdot \eta_{b}^{2}{\left({\left(v_{w}\cos\theta \right)}^{4/9}+{\left(v_{m}\sin\theta \right)}^{4/9}\right)}^{2}\cdot {\left(\frac{K_{C}^{1/2}(1-v^{2})}{2Cv_{s}C_{1}^{2}E^{7/8}}\right)}^{8/9}\cdot {\left(\tan\varphi \right)}^{2/3}H^{4/3}L_{W}R_{W}d\theta \\ \int_{-\frac{3\pi }{8}}^{-\frac{\pi }{8}}\cos\theta \cdot \frac{Y\eta_{d}^{2}}{Cv_{s}}{\left(\sqrt{v_{w}\cos\theta }+\sqrt{v_{m}\sin\theta }\right)}^{2}\tan\varphi \cdot \left(\frac{\pi }{2}-\mu_{a}\cos\varphi K_{N}\right)L_{W}R_{W}d\theta \\ \int_{-\frac{\pi }{8}}^{\frac{\pi }{8}}\cos\theta \cdot \eta_{b}^{2}{\left({\left(v_{w}\cos\theta \right)}^{4/9}+{\left(v_{m}\sin\theta \right)}^{4/9}\right)}^{2}\cdot {\left(\frac{K_{C}^{1/2}(1-v^{2})}{2Cv_{s}C_{1}^{2}E^{7/8}}\right)}^{8/9}\cdot {\left(\tan\varphi \right)}^{2/3}H^{4/3}L_{W}R_{W}d\theta \\ \int_{\frac{\pi }{8}}^{\frac{3\pi }{8}}\cos\theta \cdot \frac{Y\eta_{d}^{2}}{Cv_{s}}{\left(\sqrt{v_{w}\cos\theta }+\sqrt{v_{m}\sin\theta }\right)}^{2}\tan\varphi \cdot \left(\frac{\pi }{2}-\mu_{a}\cos\varphi K_{N}\right)L_{W}R_{W}d\theta \\ \int_{\frac{3\pi }{8}}^{\frac{\pi }{2}}\cos\theta \cdot \eta_{b}^{2}{\left({\left(v_{w}\cos\theta \right)}^{4/9}+{\left(v_{m}\sin\theta \right)}^{4/9}\right)}^{2}\cdot {\left(\frac{K_{C}^{1/2}(1-v^{2})}{2Cv_{s}C_{1}^{2}E^{7/8}}\right)}^{8/9}\cdot {\left(\tan\varphi \right)}^{2/3}H^{4/3}L_{W}R_{W}d\theta \\ \int_{\frac{\pi }{2}}^{\frac{\pi }{2}+\alpha_{b1}}\sin\left(\theta -\frac{\pi }{2}\right){\left(\frac{K_{C}^{1/2}(1-v^{2})}{2C_{1}^{2}E^{7/8}}\right)}^{8/9}\cdot {\left(\tan\varphi \right)}^{2/3}H^{4/3}{\left(\frac{v_{w}}{Cv_{s}}\right)}^{8/9}L_{W}R_{W}d\theta \end{array}\right.$$

(36) $$F_{T}=\left\{\begin{array}{l} \int_{-\frac{\pi }{2}-\alpha_{b2}}^{-\frac{\pi }{2}}\frac{\cot\varphi +\mu_{a}K_{T}}{\frac{\pi }{2}\sin\varphi -\mu_{a}\sin\varphi \cos\varphi K_{N}}{\left(\frac{K_{C}^{1/2}(1-v^{2})}{2C_{1}^{2}E^{7/8}}\right)}^{8/9}\cdot {\left(\tan\varphi \right)}^{2/3}H^{4/3}{\left(\frac{v_{w}}{Cv_{s}}\right)}^{8/9}L_{W}R_{W}d\theta \\ \int_{-\frac{\pi }{2}}^{-\frac{3\pi }{8}}\frac{\cot\varphi +\mu_{a}K_{T}}{\frac{\pi }{2}\sin\varphi -\mu_{a}\sin\varphi \cos\varphi K_{N}}\eta_{b}^{2}{\left({\left(v_{w}\cos\theta \right)}^{4/9}+{\left(v_{m}\sin\theta \right)}^{4/9}\right)}^{2}\cdot {\left(\frac{K_{C}^{1/2}(1-v^{2})}{2Cv_{s}C_{1}^{2}E^{7/8}}\right)}^{8/9}\cdot {\left(\tan\varphi \right)}^{2/3}H^{4/3}L_{W}R_{W}d\theta \\ \int_{-\frac{3\pi }{8}}^{-\frac{\pi }{8}}\frac{Y\eta_{d}^{2}}{Cv_{s}}{\left(\sqrt{v_{w}\cos\theta }+\sqrt{v_{m}\sin\theta }\right)}^{2}\tan\varphi \cdot \left(\cot\varphi +\frac{\mu_{a}K_{T}}{\sin\varphi }\right)L_{W}R_{W}d\theta \\ \int_{-\frac{\pi }{8}}^{\frac{\pi }{8}}\frac{\cot\varphi +\mu_{a}K_{T}}{\frac{\pi }{2}\sin\varphi -\mu_{a}\sin\varphi \cos\varphi K_{N}}\eta_{b}^{2}{\left({\left(v_{w}\cos\theta \right)}^{4/9}+{\left(v_{m}\sin\theta \right)}^{4/9}\right)}^{2}\cdot {\left(\frac{K_{C}^{1/2}(1-v^{2})}{2Cv_{s}C_{1}^{2}E^{7/8}}\right)}^{8/9}\cdot {\left(\tan\varphi \right)}^{2/3}H^{4/3}L_{W}R_{W}d\theta \\ \int_{\frac{\pi }{8}}^{\frac{3\pi }{8}}\frac{Y\eta_{d}^{2}}{Cv_{s}}{\left(\sqrt{v_{w}\cos\theta }+\sqrt{v_{m}\sin\theta }\right)}^{2}\tan\varphi \cdot \left(\cot\varphi +\frac{\mu_{a}K_{T}}{\sin\varphi }\right)L_{W}R_{W}d\theta \\ \int_{\frac{3\pi }{8}}^{\frac{\pi }{2}}\frac{\cot\varphi +\mu_{a}K_{T}}{\frac{\pi }{2}\sin\varphi -\mu_{a}\sin\varphi \cos\varphi K_{N}}\eta_{b}^{2}{\left({\left(v_{w}\cos\theta \right)}^{4/9}+{\left(v_{m}\sin\theta \right)}^{4/9}\right)}^{2}\cdot {\left(\frac{K_{C}^{1/2}(1-v^{2})}{2Cv_{s}C_{1}^{2}E^{7/8}}\right)}^{8/9}\cdot {\left(\tan\varphi \right)}^{2/3}H^{4/3}L_{W}R_{W}d\theta \\ \int_{\frac{\pi }{2}}^{\frac{\pi }{2}+\alpha_{b1}}\frac{\cot\varphi +\mu_{a}K_{T}}{\frac{\pi }{2}\sin\varphi -\mu_{a}\sin\varphi \cos\varphi K_{N}}{\left(\frac{K_{C}^{1/2}(1-v^{2})}{2C_{1}^{2}E^{7/8}}\right)}^{8/9}\cdot {\left(\tan\varphi \right)}^{2/3}H^{4/3}{\left(\frac{v_{w}}{Cv_{s}}\right)}^{8/9}L_{W}R_{W}d\theta \end{array}\right.$$

**Figure 10:**
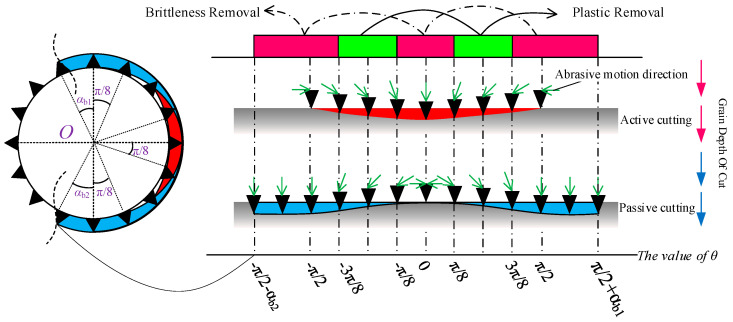
Schematic diagram of abrasive cutting depth under different cutting methods.

Equations (35) and (36) include the abrasive depth coefficients *η*_b_ and *η*_d_ corresponding to brittle and plastic removal, respectively, during wire saw cutting of loaded coal bodies. These coefficients vary with θ. Because the cutting force per abrasive grain cannot be directly determined from Equations (35) and (36), further discussion of the values of *η*_b_ and *η*_d_ in different cutting regions is required, as shown in Figure 10.

The transition criterion from plastic removal to brittle removal is given by Equation (8). When active and passive cutting act simultaneously, the brittle removal range is expanded. Within the active cutting range, brittle removal accounts for 1/5 to 1/4 of the total removal. Based on the value of 1/4 adopted above, the mutual enhancement effect between active and passive removal on cutting depth is considered negligible due to the influence of abrasive particle motion direction. Therefore, the depth coefficients *η*_a_ and *η*_b_ are both taken as 1 in the calculation. Equations (35) and (36) can then be simplified as:

(37) $$F_{N}=\left\{\begin{array}{l} \int_{-\frac{\pi }{2}-\alpha_{b2}}^{-\frac{\pi }{2}}\sin\left(\theta -\frac{\pi }{2}\right){\left(\frac{K_{C}^{1/2}(1-v^{2})}{2C_{1}^{2}E^{7/8}}\right)}^{8/9}\cdot {\left(\tan\varphi \right)}^{2/3}H^{4/3}{\left(\frac{v_{w}}{Cv_{s}}\right)}^{8/9}L_{W}R_{W}d\theta \\ \int_{-\frac{\pi }{2}}^{-\frac{3\pi }{8}}\cos\theta \cdot {\left(v_{m}\sin\theta \right)}^{8/9}\cdot {\left(\frac{K_{C}^{1/2}(1-v^{2})}{2Cv_{s}C_{1}^{2}E^{7/8}}\right)}^{8/9}\cdot {\left(\tan\varphi \right)}^{2/3}H^{4/3}L_{W}R_{W}d\theta \\ \int_{-\frac{3\pi }{8}}^{-\frac{\pi }{4}}\cos\theta \sin\theta \cdot \frac{Yv_{m}}{Cv_{s}}\tan\varphi \cdot \left(\frac{\pi }{2}-\mu_{a}\cos\varphi K_{N}\right)L_{W}R_{W}d\theta \\ \int_{-\frac{\pi }{4}}^{-\frac{\pi }{8}}\cos^{2}\theta \cdot \frac{Yv_{m}}{Cv_{s}}\tan\varphi \cdot \left(\frac{\pi }{2}-\mu_{a}\cos\varphi K_{N}\right)L_{W}R_{W}d\theta \\ \int_{-\frac{\pi }{8}}^{-\frac{\pi }{8}}\cos\theta \cdot {\left(v_{m}\sin\theta \right)}^{8/9}\cdot {\left(\frac{K_{C}^{1/2}(1-v^{2})}{2Cv_{s}C_{1}^{2}E^{7/8}}\right)}^{8/9}\cdot {\left(\tan\varphi \right)}^{2/3}H^{4/3}L_{W}R_{W}d\theta \\ \int_{\frac{\pi }{8}}^{\frac{\pi }{4}}\cos^{2}\theta \cdot \frac{Yv_{m}}{Cv_{s}}\tan\varphi \cdot \left(\frac{\pi }{2}-\mu_{a}\cos\varphi K_{N}\right)L_{W}R_{W}d\theta \\ \int_{\frac{\pi }{4}}^{\frac{3\pi }{8}}\cos\theta \sin\theta \cdot \frac{Yv_{m}}{Cv_{s}}\tan\varphi \cdot \left(\frac{\pi }{2}-\mu_{a}\cos\varphi K_{N}\right)L_{W}R_{W}d\theta \\ \int_{\frac{3\pi }{8}}^{\frac{\pi }{2}}\cos\theta \cdot {\left(v_{m}\sin\theta \right)}^{8/9}\cdot {\left(\frac{K_{C}^{1/2}(1-v^{2})}{2Cv_{s}C_{1}^{2}E^{7/8}}\right)}^{8/9}\cdot {\left(\tan\varphi \right)}^{2/3}H^{4/3}L_{W}R_{W}d\theta \\ \int_{\frac{\pi }{2}}^{\frac{\pi }{2}+\alpha_{b1}}\sin\left(\theta -\frac{\pi }{2}\right){\left(\frac{K_{C}^{1/2}(1-v^{2})}{2C_{1}^{2}E^{7/8}}\right)}^{8/9}\cdot {\left(\tan\varphi \right)}^{2/3}H^{4/3}{\left(\frac{v_{w}}{Cv_{s}}\right)}^{8/9}L_{W}R_{W}d\theta \end{array}\right.$$

(38) $$F_{T}=\left\{\begin{array}{l} \int_{-\frac{\pi }{2}-\alpha_{b2}}^{-\frac{\pi }{2}}\frac{\cot\varphi +\mu_{a}K_{T}}{\frac{\pi }{2}\sin\varphi -\mu_{a}\sin\varphi \cos\varphi K_{N}}{\left(\frac{K_{C}^{1/2}(1-v^{2})}{2C_{1}^{2}E^{7/8}}\right)}^{8/9}\cdot {\left(\tan\varphi \right)}^{2/3}H^{4/3}{\left(\frac{v_{w}}{Cv_{s}}\right)}^{8/9}L_{W}R_{W}d\theta \\ \int_{-\frac{\pi }{2}}^{-\frac{3\pi }{8}}\frac{\cot\varphi +\mu_{a}K_{T}}{\frac{\pi }{2}\sin\varphi -\mu_{a}\sin\varphi \cos\varphi K_{N}}{\left(v_{m}\sin\theta \right)}^{8/9}\cdot {\left(\frac{K_{C}^{1/2}(1-v^{2})}{2Cv_{s}C_{1}^{2}E^{7/8}}\right)}^{8/9}\cdot {\left(\tan\varphi \right)}^{2/3}H^{4/3}L_{W}R_{W}d\theta \\ \int_{-\frac{3\pi }{8}}^{-\frac{\pi }{4}}\frac{Yv_{m}}{Cv_{s}}\sin\theta \tan\varphi \cdot \left(\cot\varphi +\frac{\mu_{a}K_{T}}{\sin\varphi }\right)L_{W}R_{W}d\theta \\ \int_{-\frac{\pi }{4}}^{-\frac{\pi }{8}}\frac{Yv_{m}}{Cv_{s}}\cos\theta \tan\varphi \cdot \left(\cot\varphi +\frac{\mu_{a}K_{T}}{\sin\varphi }\right)L_{W}R_{W}d\theta \\ \int_{-\frac{\pi }{8}}^{\frac{\pi }{8}}\frac{\cot\varphi +\mu_{a}K_{T}}{\frac{\pi }{2}\sin\varphi -\mu_{a}\sin\varphi \cos\varphi K_{N}}{\left(v_{w}\cos\theta \right)}^{8/9}\cdot {\left(\frac{K_{C}^{1/2}(1-v^{2})}{2Cv_{s}C_{1}^{2}E^{7/8}}\right)}^{8/9}\cdot {\left(\tan\varphi \right)}^{2/3}H^{4/3}L_{W}R_{W}d\theta \\ \int_{\frac{\pi }{8}}^{\frac{\pi }{4}}\frac{Yv_{m}}{Cv_{s}}\cos\theta \tan\varphi \cdot \left(\cot\varphi +\frac{\mu_{a}K_{T}}{\sin\varphi }\right)L_{W}R_{W}d\theta \\ \int_{\frac{\pi }{4}}^{\frac{3\pi }{8}}\frac{Yv_{m}}{Cv_{s}}\sin\theta \tan\varphi \cdot \left(\cot\varphi +\frac{\mu_{a}K_{T}}{\sin\varphi }\right)L_{W}R_{W}d\theta \\ \int_{\frac{3\pi }{8}}^{\frac{\pi }{2}}\frac{\cot\varphi +\mu_{a}K_{T}}{\frac{\pi }{2}\sin\varphi -\mu_{a}\sin\varphi \cos\varphi K_{N}}{\left(v_{m}\sin\theta \right)}^{8/9}\cdot {\left(\frac{K_{C}^{1/2}(1-v^{2})}{2Cv_{s}C_{1}^{2}E^{7/8}}\right)}^{8/9}\cdot {\left(\tan\varphi \right)}^{2/3}H^{4/3}L_{W}R_{W}d\theta \\ \int_{\frac{\pi }{2}}^{\frac{\pi }{2}+\alpha_{b1}}\frac{\cot\varphi +\mu_{a}K_{T}}{\frac{\pi }{2}\sin\varphi -\mu_{a}\sin\varphi \cos\varphi K_{N}}{\left(\frac{K_{C}^{1/2}(1-v^{2})}{2C_{1}^{2}E^{7/8}}\right)}^{8/9}\cdot {\left(\tan\varphi \right)}^{2/3}H^{4/3}{\left(\frac{v_{w}}{Cv_{s}}\right)}^{8/9}L_{W}R_{W}d\theta \end{array}\right.$$

Applying symmetry relationships and integrating Equations (37) and (38) yields:

(39) $$F_{N}=\left\{\begin{array}{l} (1-\cos\alpha_{b2})A_{NT}{\left(\frac{v_{w}}{Cv_{s}}\right)}^{8/9}L_{W}R_{W},\left(-\frac{\pi }{2}-\alpha_{b2},-\frac{\pi }{2}\right)\\ 0.7285\cdot A_{NT}{\left(\frac{v_{w}}{Cv_{s}}\right)}^{8/9}L_{W}R_{W},\left(-\frac{\pi }{8},\frac{\pi }{8}\right)\\ 0.2996\cdot B_{NT}\frac{Yv_{m}}{Cv_{s}}L_{W}R_{W},\left(-\frac{\pi }{4},-\frac{\pi }{8}\right)\cup \left(\frac{\pi }{8},\frac{\pi }{4}\right)\\ 0.1768\cdot B_{NT}\frac{Yv_{m}}{Cv_{s}}L_{W}R_{W},\left(-\frac{3\pi }{8},-\frac{\pi }{4}\right)\cup \left(\frac{\pi }{4},\frac{3\pi }{8}\right)\\ 0.0734\cdot A_{NT}{\left(\frac{v_{w}}{Cv_{s}}\right)}^{8/9}L_{W}R_{W},\left(-\frac{\pi }{2},-\frac{3\pi }{8}\right)\cup \left(\frac{3\pi }{8},\frac{\pi }{2}\right)\\ (1-\cos\alpha_{b1})A_{NT}{\left(\frac{v_{w}}{Cv_{s}}\right)}^{8/9}L_{W}R_{W},\left(\frac{\pi }{2},\frac{\pi }{2}+\alpha_{b2}\right) \end{array}\right.$$

(40) $$F_{T}=\left\{\begin{array}{l} 0.7620\cdot C_{NT}A_{NT}{\left(\frac{v_{w}}{Cv_{s}}\right)}^{8/9}L_{W}R_{W},\left(-\frac{\pi }{8},\frac{\pi }{8}\right)\\ 0.3244\cdot D_{NT}\frac{v_{m}}{Cv_{s}}L_{W}R_{W},\left(-\frac{\pi }{4},-\frac{\pi }{8}\right)\cup \left(\frac{\pi }{8},\frac{\pi }{4}\right)\\ 0.3244\cdot D_{NT}\frac{v_{m}}{Cv_{s}}L_{W}R_{W},\left(-\frac{3\pi }{8},-\frac{\pi }{4}\right)\cup \left(\frac{\pi }{4},\frac{3\pi }{8}\right)\\ 0.3821\cdot C_{NT}A_{NT}{\left(\frac{v_{w}}{Cv_{s}}\right)}^{8/9}L_{W}R_{W},\left(-\frac{\pi }{2},-\frac{3\pi }{8}\right)\cup \left(\frac{3\pi }{8},\frac{\pi }{2}\right)\\ C_{NT}A_{NT}{\left(\frac{v_{w}}{Cv_{s}}\right)}^{8/9}L_{W}R_{W},\left(-\frac{\pi }{2}-\alpha_{b2},-\frac{\pi }{2}\right)\cup \left(\frac{\pi }{2},\frac{\pi }{2}+\alpha_{b2}\right) \end{array}\right.$$

In the formula, $A_{NT}={\left(\frac{K_{C}^{1/2}(1-v^{2})}{2Cv_{s}C_{1}^{2}E^{7/8}}\right)}^{8/9}\cdot {\left(\tan\varphi \right)}^{2/3}H^{4/3}$, $B_{NT}=\tan\varphi \cdot \left(\frac{\pi }{2}-\mu_{a}\cos\varphi K_{N}\right)$, $C_{NT}=\frac{\cot\varphi +\mu_{a}K_{T}}{\frac{\pi }{2}\sin\varphi -\mu_{a}\sin\varphi \cos\varphi K_{N}}$, $D_{NT}=Y\tan\varphi \cdot \left(\cot\varphi +\frac{\mu_{a}K_{T}}{\sin\varphi }\right)$. The values of these coefficients are all related to tool characteristics, cutting material properties, and cooling or lubrication conditions.

### 2.3. Cutting Force Model for Wire Saws in the Cutting Zone

**Figure 11:**
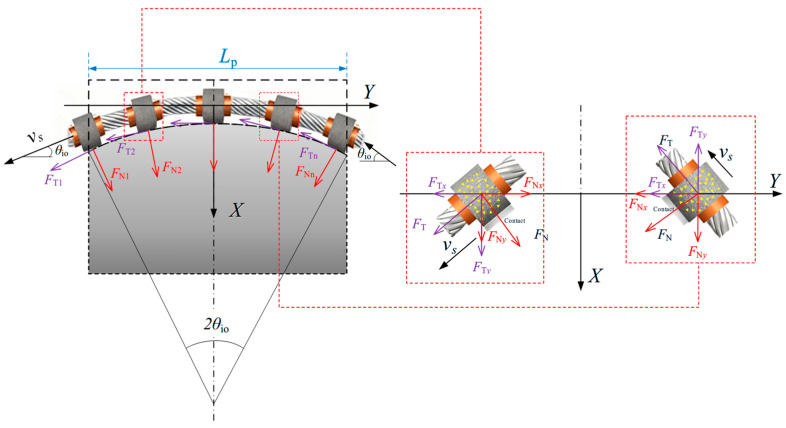
Schematic diagram of force distribution on different beads in the contact zone.

During coal cutting, the wire saw forms an arc-shaped contact zone with the coal body, as shown in Figure 11. The tangential cutting force exerted by each bead on the coal body is aligned with the movement direction of the wire saw, while the normal cutting force is perpendicular to the movement direction. Analysis shows that the vertical cutting force exerted by the wire saw on the processed coal body is the resultant of the component forces in the negative *X*-direction from all beads along the cutting path within the arc zone. The horizontal cutting force exerted by the wire saw on the processed coal body is the resultant of the component forces in the negative *Y*-direction along the vertical cutting path.

In calculating the cutting force over the entire cutting zone, it is assumed that the entry and exit angles of the wire saw are both *θ*_io_, and that the length of the cut coal body is *L*_p_. The corresponding arc length *L*_AC_ of the cutting zone is:

(41) $$L_{AC}=\frac{\theta_{io}L_{p}\pi }{180}\sin^{-1}\theta_{io}$$

Let the arc length parameter be *s*, where s ranges from *s* = 0 to *s* = *L*_AC_. The force density per unit length along the arc has a tangential component *T*_s_ and a normal component *N*_s_. Let *θ*(s) denote the angle between the tangential direction and the *Y*-axis, i.e., the tangential angle.

Accordingly, the normal and tangential force density components can be expressed as Equations (42) and (43), respectively.

(42) $$\left\{\begin{array}{l} f_{n}^{X}(s)=N_{s}\cos\theta (s)\\ f_{n}^{Y}(s)=-N_{s}\sin\theta (s) \end{array}\right.$$

(43) $$\left\{\begin{array}{l} f_{t}^{X}(s)=T_{s}\cos\theta (s)\\ f_{t}^{Y}(s)=-T_{s}\sin\theta (s) \end{array}\right.$$

Based on Equations (42) and (43), the resultant force density component can be further expressed as:

(44) $$\left\{\begin{array}{l} f_{X}(s)=T_{s}\sin\theta (s)+N_{s}\cos\theta (s)\\ f_{Y}(s)=T_{s}\cos\theta (s)-N_{s}\sin\theta (s) \end{array}\right.$$

Thus, the vertical and horizontal cutting forces exerted by the wire saw on the loaded coal body can be obtained as:

(45) $$\left\{\begin{array}{l} F_{X}=\int_{0}^{L_{AC}}f_{X}(s)ds=T_{s}\int_{0}^{L_{AC}}\sin\theta (s)ds+N_{s}\int_{0}^{L_{AC}}\sin\theta (s)ds\\ F_{Y}=\int_{0}^{L_{AC}}f_{Y}(s)ds=T_{s}\int_{0}^{L_{AC}}\cos\theta (s)ds-N_{s}\int_{0}^{L_{AC}}\sin\theta (s)ds \end{array}\right.$$

Using the geometric relationships shown in Figure 9, Equation (45) can be rewritten as Equation (46), specifically as follows:

(46) $$\left\{\begin{array}{l} F_{X}=T_{s}△X-N_{s}△Y\\ F_{Y}=T_{s}△Y-N_{s}△X \end{array}\right.$$

In the formula, (△*X*, △*Y*) represents the coordinate difference between the start point and end point of the arc.

When calculating the cutting force acting on the loaded coal body, the force exerted by each bead can be treated as a concentrated load. The vertical and horizontal cutting forces on the loaded coal body can therefore be expressed as:

(47) $$\left\{\begin{array}{l} F_{NN}=(F_{T}△X-F_{N}△Y)n_{d}=-F_{N}△Yn_{d}=-F_{N}L_{p}n_{d}\\ F_{TT}=(F_{T}△Y-F_{N}△X)n_{d}=F_{T}△Yn_{d}=F_{T}L_{p}n_{d} \end{array}\right.$$

In this equation, *n*_d_ represents the number of beads per unit length of the wire saw, i.e., the bead density.

Substituting the normal and tangential cutting forces per abrasive particle obtained from Equations (29), (39) and (40) into Equation (47) yields the cutting force acting on the loaded coal body. Owing to the excessive length of the resulting expression, it is not expanded here. Analysis of Equation (47) indicates that the cutting force during wire saw cutting of a loaded coal body is influenced by multiple factors, including wire saw cutting conditions, coal seam properties, and process parameters. Increasing the load changes the cutting depth of individual abrasive grains in the coal body, converting plastic removal in the coal regions adjacent to each bead (3π/8 to π/2 and –π/2 to –3π/8) into brittle removal. At the same time, the brittle cutting range extends beyond π/2 and –π/2. The combined effect of these changes alters the cutting force acting on the loaded coal body during wire saw cutting.

Considering that the same type of wire saw is generally used under similar operating conditions in practical cutting operations, the cutting force exerted by a single bead on a given coal seam mainly depends on the wire saw linear velocity *v*_s_, feed rate *v*_w_, and wire saw radius *R*_w_.

## 3. Wire Saw Cutting Experiment on Loaded Coal Body

### 3.1. Wire Saw Cutting Experiment Apparatus for Loaded Coal Body

At present, the underground cutting of loaded coal seams using wire saw technology has not been widely applied, and the development of corresponding equipment remains limited, with most studies restricted to indoor experimental research. Based on the structural characteristics of wire saw cutting in loaded coal bodies, and building upon an indirect cutting force measurement device for wire saw cutting of loaded coal bodies [31], a wire saw cutting test apparatus for loaded coal bodies was further designed. This apparatus mainly consists of three parts: an elevatable wire saw cutting system, a horizontal loading device, and a cutting force data acquisition system. The wire saw cutting system includes a synchronous lifting platform, a variable-speed motor, a diamond bead wire, and a tensioning pulley. The synchronous lifting platform controls the cutting feed rate, while the variable-speed motor adjusts the cutting line speed of the wire saw. The diamond bead wire can be replaced with different diameters as required, and the tensioning pulley maintains the initial tension during cutting to prevent wire slippage.

**Figure 12:**
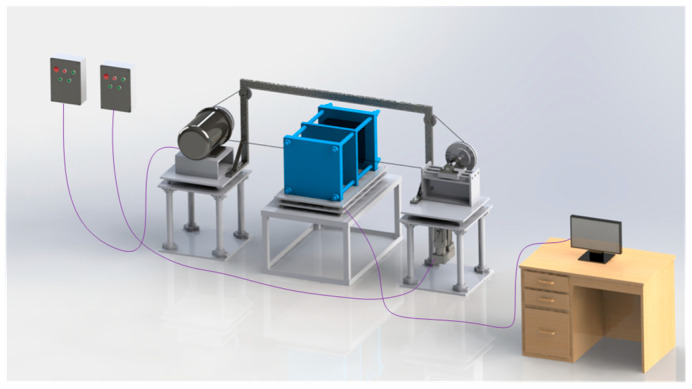
Overall assembly drawing of the wire saw cutting experimental apparatus for loaded coal.

**Figure 13:**
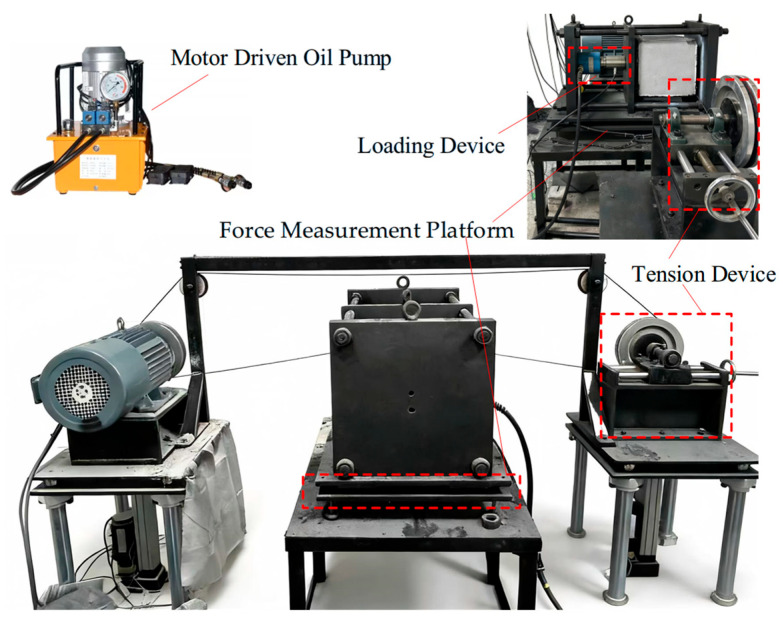
Actual photo of the wire saw cutting experimental apparatus for loaded coal mass.

The horizontal loading device is used to apply uniaxial compression to standard specimens with dimensions of 300 mm × 300 mm × 300 mm. A high-elasticity polycarbonate (PC) plate is introduced as a buffer transition component between the loading surface of the device and the specimen contact surface. This configuration allows elastic deformation of the PC plate to release constraints after the wire saw forms a cut, thereby partially simulating the mechanical stress response under engineering conditions. The overall structure and the actual apparatus are shown in Figure 12 and Figure 13.

Based on the above design, the main technical parameters of the wire saw cutting experimental apparatus for loaded coal mass are as follows: (1) wire saw linear speed: 0–20 m/s; (2) wire saw feed rate: 0–20 mm/min; (3) horizontal loading force: 0–500 kN; (4) tension sensor measurement range: 0–5 kN; (5) Kistler 9257B three-dimensional force measurement platform: horizontal and vertical cutting force range of 0–1 kN, with a sampling frequency of 1–40 Hz.

### 3.2. Experimental Protocol

**Figure 14:**
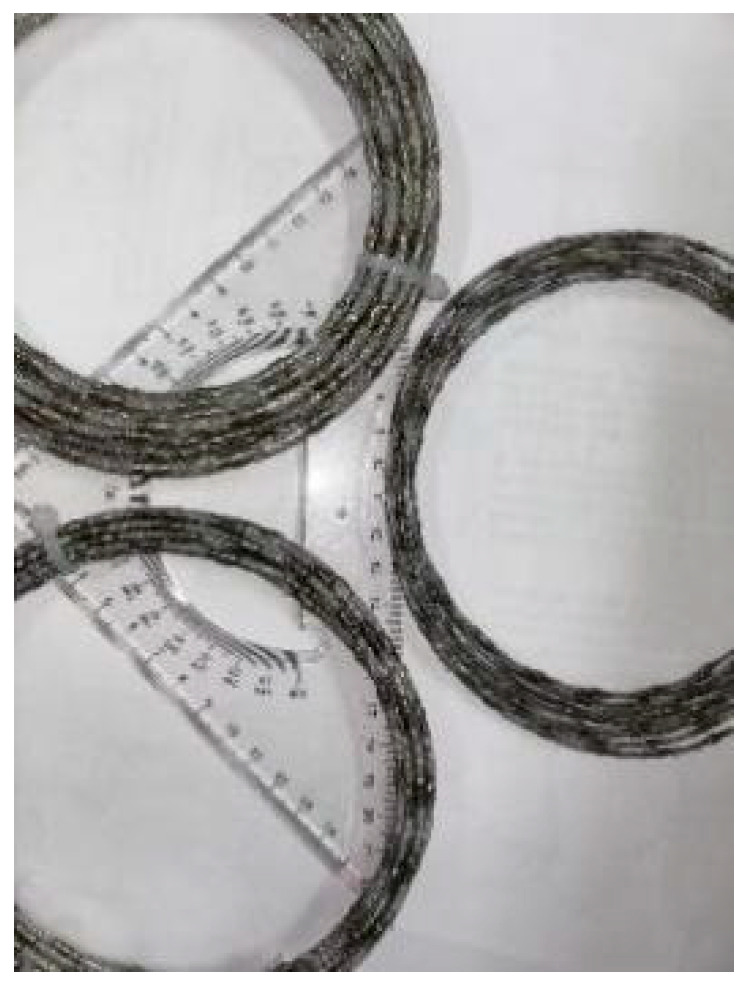
Physical image of brazed wire saw.

A brazed wire saw was used to cut the loaded coal body in the experiment, and its physical appearance is shown in Figure 14. The main technical parameters of the wire saw are listed in Table 1.

During validation of the cutting force model, the influence of coal body damage on cutting force needed to be minimized; therefore, briquettes with relatively higher strength were selected. The main performance parameters of the loaded coal body used in the cutting experiments are shown in Table 2.

To compare the variation in cutting force before and after coal loading during wire cutting, a blank control group without applied load was established. The cutting parameters for the loaded coal experiments are listed in Table 3. To avoid safety risks caused by wire disengagement during cutting, the initial tension of the wire saw was set to *T*_0_ = 200 N.

Before the experiment, the coal surface was coated with white matte paint, followed by the application of speckle markers. Digital image correlation (DIC) was used to obtain the displacement distribution on the coal surface during the cutting process. At the same time, laser scanning was performed on the cut coal surface to compare slit depths and determine changes in coal removal modes before and after loading during wire saw cutting. Cutting force acquisition was terminated once both vertical and horizontal cutting forces reached a stable state, with a sampling frequency of 5 Hz.

### 3.3. Results and Analysis

**Figure 15:**
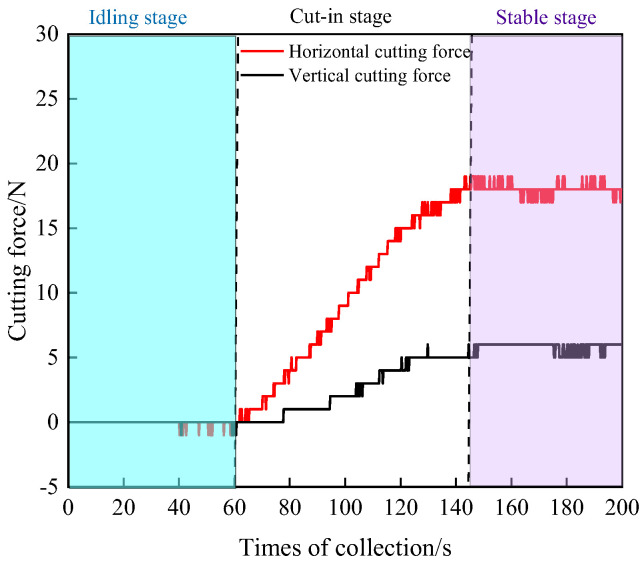
Cutting force signal curve under non-loaded conditions.

Figure 15 shows the cutting force signal curve obtained during wire saw cutting of coal under non-loaded conditions. The cutting process can be divided into three stages: the no-load stage, the penetration stage, and the stable cutting stage. From observation of the cutting process, during the initial contact stage, owing to the flexible machining characteristics of the wire saw, the wire saw bends at both ends of the coal specimen as it advances downward, generating contact pressure. This pressure initiates cutting at both ends of the specimen. As cutting continues, the contact area between the wire saw and the coal body gradually increases until the wire saw fully penetrates the specimen, forming a stable cutting arc zone within the coal body.

**Figure 16:**
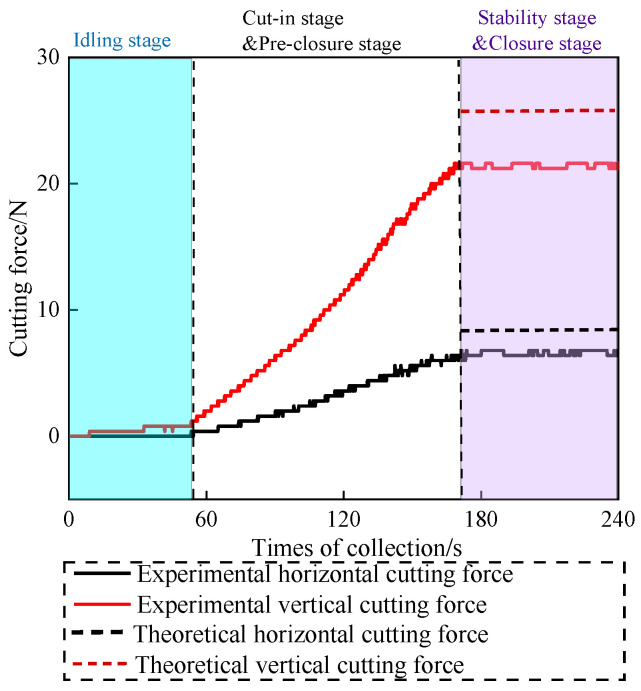
Cutting force signal curve under loaded conditions.

**Figure 17:**
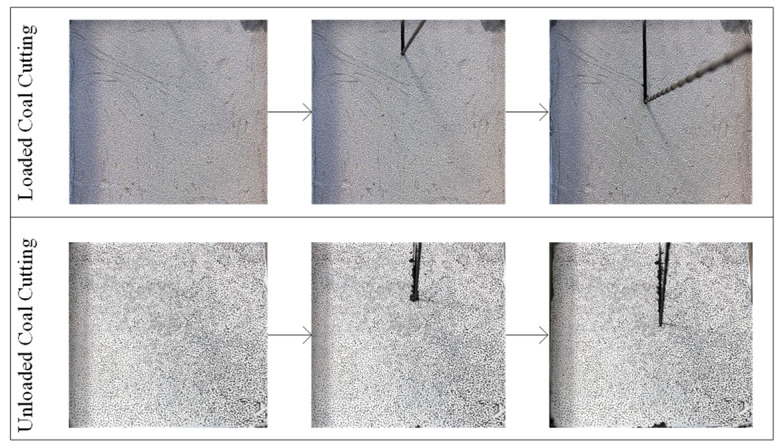
Cutting process before and after loading.

**Figure 18:**
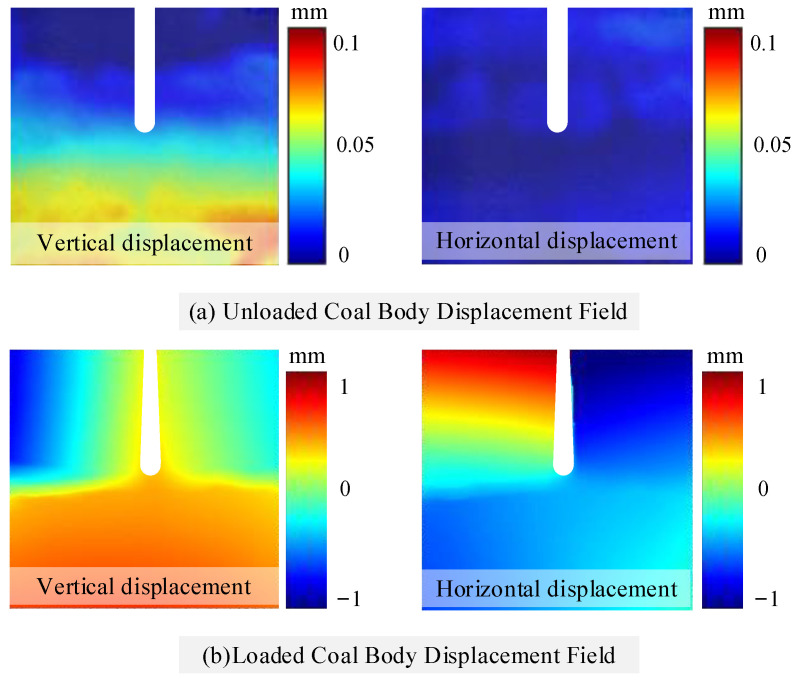
Displacement field variation in the coal body before and after loading during wire saw cutting.

Figure 16 shows the cutting force signal curve of the coal body under load during wire saw cutting. Table 4 shows the comparison between the experimental cutting force and the theoretical cutting force. Comparison with the cutting force signal curve of the unloaded specimen shows that loading enhances both the horizontal and vertical cutting forces during the stable stage of wire saw cutting. Combined with theoretical calculations, it is inferred that the forces applied to both sides of the coal body transform small-scale plastic removal into brittle removal, which typically reduces the cutting force exerted by the wire saw on the coal body. However, loading on both sides of the coal body also increases the cutting range of the wire saw, and expansion of the cutting range leads to an increase in cutting force. The results indicate that the reduction in cutting force caused by the former effect is smaller than the increase caused by the latter. During wire saw cutting, the linear speed and feed rate are controlled by a variable-speed motor and a stepper motor, respectively. Under sufficient power conditions, these parameters are not affected by loading. When the coal specimen is loaded on both sides, the coal on either side of the slit displaces inward toward the slit. Figure 17 and Figure 18 show the process of cutting coal before and after loading by wire saw, and the change in the displacement field of coal before and after cutting by wire saw. This displacement not only compresses the coal on both sides toward the abrasive grains of the wire saw but also increases the cutting depth of individual abrasive grains. This effect expands the brittle removal zone and converts part of the plastic removal into brittle removal. The combined influence of these factors results in an increase in the cutting force of the wire saw acting on the loaded coal specimen. It is also observed that, during wire saw cutting of loaded coal, the stage of increasing cutting force lasts longer, and the process enters the stable stage later. This occurs because, after the wire saw fully penetrates the loaded specimen, the coal on both sides of the slit remains under pressure, and slit closure occurs only after a certain cutting length is reached. Before closure, the passive cutting range continues to expand, causing the cutting force to increase during both the initial penetration stage and the period prior to slit closure. In theoretical cutting force calculations, certain range parameters and hardness parameters complicate the data solution. Therefore, the stable stage, which is easier to identify and measure, was selected for calculation. Table 4 shows that the relative error between the theoretical value and the experimental value is within 13.6%, and the theoretical value is greater than the experimental value. This discrepancy is attributed to the theoretical derivation not accounting for secondary factors such as lubrication. In addition, for safety considerations, all range parameters in the theoretical calculation are assumed to have a positive contribution to the cutting force.

**Figure 19:**
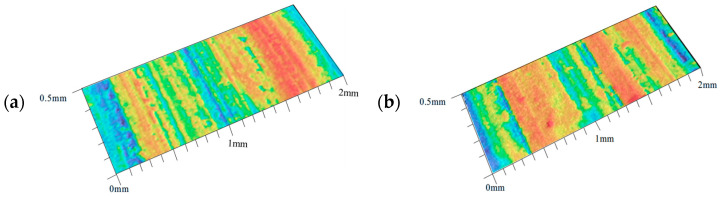
Laser scanning images of cutting surface profiles before and after loading. (**a**) Cutting surface of loaded coal body. (**b**) Cutting surface of unloaded coal body.

The surface roughness was measured using a professional surface roughness tester after the sawing experiment. Three different positions were randomly selected on each cutting surface for measurement, and the arithmetic mean roughness (Ra) and maximum height roughness (Rz) were recorded, respectively. The final values of Ra and Rz were obtained by taking the average of three repeated measurements to ensure accuracy and reliability. After cutting loaded and unloaded coal bodies, the contour profiles of the cut surfaces are shown in Figure 19. The arithmetic mean roughness (Ra) of the loaded coal cutting surface is 31.96 μm, and the maximum height roughness (Rz) is 169.14 μm. Both values are higher than those of the unloaded coal cutting surface, which exhibits a Ra of 23.93 μm and an Rz of 118.15 μm. The arithmetic mean roughness Ra represents the average of the absolute deviations of the surface profile from the mean line over the evaluation length and reflects the overall surface condition. These results indicate that, after cutting under loading, the average surface roughness of the coal body increases, and the cutting process generates a larger number of deeper micro-fractures and slits. The maximum height roughness Rz represents the vertical distance between the highest peak and the lowest valley within the evaluation length and reflects the most severe surface defects. After loading on both sides of the coal body, the “plow effect” becomes more pronounced. Some protruding abrasive grains or grain clusters on the wire saw may slide along the surface under pressure, producing intense plowing and pushing actions that force material toward both sides and form higher ridges, corresponding to higher surface peaks. Overall, the cutting surface of loaded coal compressed from both sides becomes rougher and more uneven, and the cutting mechanism transitions from relatively “smooth grinding” to “violent brittle fracture”.

## 4. Conclusions

The loading applied to both sides of the coal block increases the effective cutting depth of the abrasive grains, causing the material removal mechanism to shift from plastic to brittle cutting. This effect is primarily concentrated in the ranges of 3π/8 to π/2 and −π/2 to −3π/8 along the string; simultaneously, the loading expands the brittle cutting range between the wire saw and the coal block in the regions beyond π/2 and within −π/2.

The shift in the abrasive particle removal mode toward high-efficiency brittle cutting helps reduce the cutting force, whereas the expansion of the cutting contact area increases the cutting force. Experiments indicate that the increase in cutting force resulting from the expanded cutting range outweighs the reduction effect generated by the optimized removal mode.

The phase of rising cutting force in the loaded coal body is longer, and the stabilization phase is delayed, due to the continuous expansion of the passive cutting area before the slit closes under pressure; the surface roughness of the cutting surface is significantly higher than that of the unloaded coal body, indicating a transition from smooth grinding to violent brittle fracture, with intensified micro-fracturing and plowing effects.

Based on the three-dimensional cutting force model established in this study, future research can be further deepened in five priority aspects: First, introduce triaxial stress conditions to accurately characterize the slit closure effect combined with the real stress field. Second, clarify the fracture and failure behavior of the loaded coal mass and its mechanism affecting the wire saw cutting force, so as to optimize the existing model. Third, systematically analyze the impacts of abrasive particle shape, interfacial friction coefficient and other parameters on the cutting process to improve the model’s applicability under complex working conditions. Fourth, incorporate the abrasive wear effect into the model framework to enhance its prediction accuracy in actual engineering scenarios.

## Figures and Tables

**Table 1:** Key technical specifications of wire saws.

Name	Parameter
Abrasive grain hemi-cone angle *φ*/(°)	55
Abrasive grain density *C*/mm^−2^	40
Single bead length *L*_W_/mm	6.5
Bead radius *R*_W_/mm	4.8
Total beads per unit length *n*_d_/m^−1^	37

**Table 2:** Key performance indicators of the loaded coal specimen used in experiment.

Name	Parameter
Microhardness *H*/GPa	0.115
Elastic Modulus *E*/GPa	4.2
Poisson’s Ratio *v*	0.26
Relative Plastic Dimension *β*	3.63
Length × Width × Height/mm × mm × mm	300 × 300 × 300
Fracture Toughness *K*_C_/MPa·m^1/2^	1.18

**Table 3:** Experiment cutting parameter settings.

Name	Parameter
Wire saw linear speed *v*_s_/m·s^−1^	15
Wire saw feed speed *v*_w_/mm·min^−1^	1
Passive cutting velocity *v*_m_/mm·min^−1^	0.5
Load applied to specimen/kN	200
Guide wheel diameter *D*/m	200 mm

**Table 4:** Comparison of experimental cutting force and theoretical cutting force.

The Direction of Cutting Force	Theoretical Cutting Force/N	Experimental Cutting Force/N	Relative Error Value
Vertical cutting force	24.23	21.40	11.7%
Horizontal cutting force	7.64	6.60	13.6%

## Data Availability

The original contributions presented in this study are included in the article. Further inquiries can be directed to the corresponding authors.
